# Ibrutinib-induced pneumonitis in chronic lymphocytic leukemia patient

**DOI:** 10.5339/qmj.2024.qitc.15

**Published:** 2024-03-25

**Authors:** Salem Salah

**Affiliations:** 1 Hamad General Hospital, Doha, Qatar Email: sabosalah@hamad.qa

**Keywords:** Ibrutinib, Ild, Pneumonitis, Drug Induced

## Introduction

Ibrutinib, a widely used Bruton tyrosine kinase inhibitor, is extensively used in the treatment of lymphocytic malignancies, including chronic lymphocytic leukemia (CLL).^1,2^ Respiratory complications associated with the use of ibrutinib include cough (13–22%), dyspnea (10–12%), oropharyngeal pain (14%), pneumonia (12–23%), sinusitis (11–22%), upper respiratory tract infection (16–47%), pneumonitis, and interstitial lung disease.^3^

## Case Presentation

A 71-year-old male diagnosed with CLL and receiving ibrutinib for three years presented a challenging clinical scenario. Although he was admitted to the hospital three times for pneumonitis, his symptoms persisted and imaging indicated a worsening condition. Over a span of four months, he experienced a persistent cough and shortness of breath, and eventually developed asthenia, skin dryness, and severe pruritus.

Further investigation using chest X-ray and CT scans identified interstitial lung infiltrates with ground-glass opacities, leading to the suspicion of ibrutinib-induced pneumonitis and dry pruritus. Despite the recommendation to discontinue ibrutinib, the patient was initially hesitant and refused bronchoscopy and lung biopsy. Initiation of prednisone therapy did not yield a satisfactory response until the patient agreed to discontinue ibrutinib.

Approximately two weeks after discontinuation, the patient showed remarkable improvement, with almost complete resolution of symptoms and significant improvement in chest X-ray findings, confirming the association between the use of ibrutinib and the observed pneumonitis.

## Conclusion

Suspicion of ibrutinib-induced lung injury necessitates prompt discontinuation, as failure to do so may result in the development of potentially severe and permanent interstitial lung disease. It is crucial to recognize that this lung injury can manifest itself after prolonged use of ibrutinib, even spanning several years. Instances of side effects in other organs, such as the skin changes observed in our case, should heighten awareness of the likelihood of ibrutinib-induced lung injury. Notably, the administration of steroids alone, without discontinuing the implicated medication, is insufficient to resolve this complication.

## Conflict of Interest

Author has no conflict of interest.

## Figures and Tables

**Figure 1.**
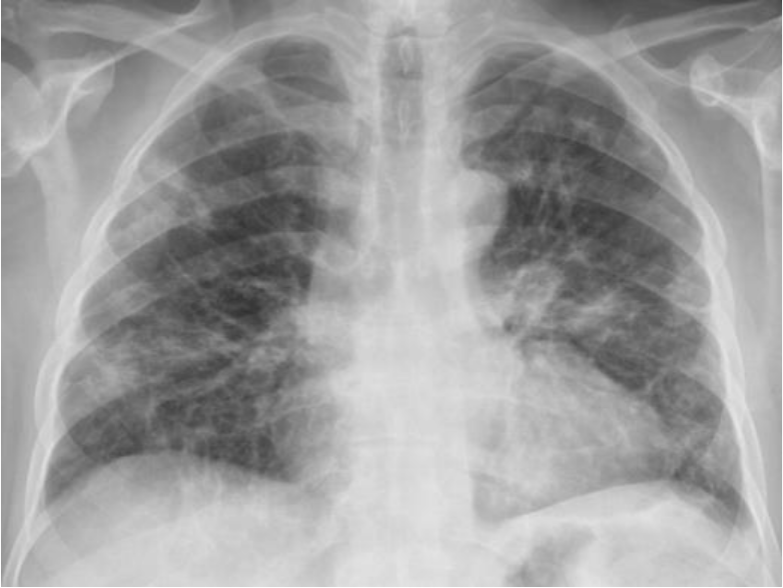
Before holding ibrutinib (12 April 2023).

**Figure 2.**
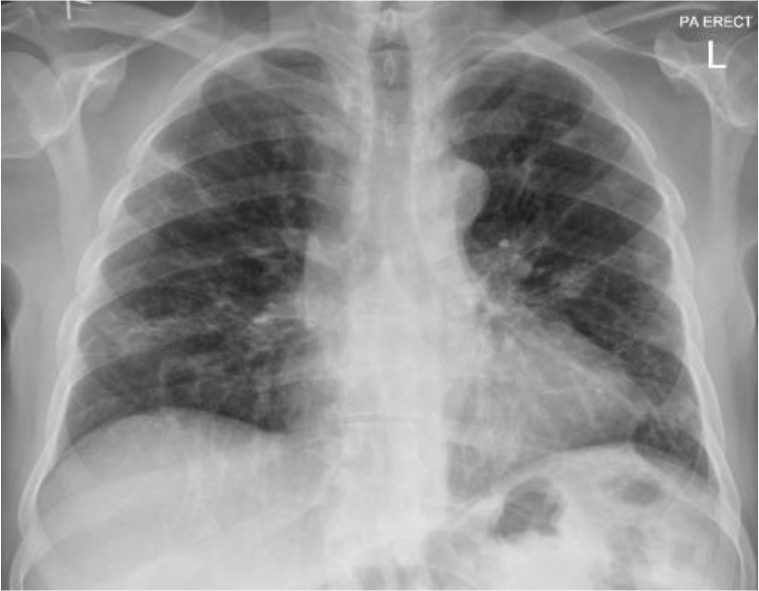
After stopping ibrutinib (22 May 2023).
